# Chelator-Free/Chelator-Mediated Radiolabeling of Colloidally Stabilized Iron Oxide Nanoparticles for Biomedical Imaging

**DOI:** 10.3390/nano11071677

**Published:** 2021-06-25

**Authors:** Sofia Papadopoulou, Argiris Kolokithas-Ntoukas, Evangelia-Alexandra Salvanou, Anastasios Gaitanis, Stavros Xanthopoulos, Konstantinos Avgoustakis, Maria Gazouli, Maria Paravatou-Petsotas, Charalampos Tsoukalas, Aristides Bakandritsos, Penelope Bouziotis

**Affiliations:** 1 Institute of Nuclear & Radiological Sciences & Technology, Energy & Safety, National Center for Scientific Research “Demokritos”, 15341 Athens, Greece; sp@radioanalytics.eu (S.P.); salvanou@rrp.demokritos.gr (E.-A.S.); staxan@rrp.demokritos.gr (S.X.); mparavatou@rrp.demokritos.gr (M.P.-P.); ctsoukal@rrp.demokritos.gr (C.T.); 2 Radioanalytics-Environmental Radioactivity, Radiochemistry & Radiobiology Research Laboratories SMPC, 20131 Corinth, Greece; 3 Department of Pharmacy, School of Health Sciences, University of Patras, 26504 Patras, Greece; kolokithas@upatras.gr (A.K.-N.); avgoust@upatras.gr (K.A.); 4 Department of Materials Science, School of Natural Sciences, University of Patras, 26504 Patras, Greece; 5 Biomedical Research Foundation of the Academy of Athens, 11527 Athens, Greece; agaitanis@bioacademy.gr; 6 Department of Basic Medical Sciences, Laboratory of Biology, Medical School, National and Kapodistrian University of Athens, 11527 Athens, Greece; mgazouli@med.uoa.gr; 7 Regional Centre of Advanced Technologies and Materials, Czech Advanced Technology and Research Institute, Palacký University, 77900 Olomouc, Czech Republic; a.bakandritsos@upol.cz; 8 Nanotechnology Centre, Centre of Energy and Environmental Technologies, VŠB–Technical University of Ostrava, 17. listopadu 2172/15, 708 00 Ostrava-Poruba, Czech Republic

**Keywords:** Gallium-68, iron oxide nanoparticles, MTT, radiolabeling, biodistribution, PET

## Abstract

The aim of this study was to develop a bioimaging probe based on magnetic iron oxide nanoparticles (MIONs) surface functionalized with the copolymer (p(MAA-g-EGMA)), which were radiolabeled with the positron emitter Gallium-68. The synthesis of the hybrid MIONs was realized by hydrolytic condensation of a single ferrous precursor in the presence of the copolymer. The synthesized MagP MIONs displayed an average D_h_ of 87 nm, suitable for passive targeting of cancerous tissues through the enhanced permeation and retention (EPR) effect after intravenous administration, while their particularly high magnetic content ascribes strong magnetic properties to the colloids. Two different approaches were explored to develop MIONs radiolabeled with ^68^Ga: the chelator-mediated approach, where the chelating agent NODAGA-NHS was conjugated onto the MIONs (MagP-NODAGA) to form a chelate complex with ^68^Ga, and the chelator-free approach, where ^68^Ga was directly incorporated onto the MIONs (MagP). Both groups of NPs showed highly efficient radiolabeling with ^68^Ga, forming constructs which were stable with time, and in the presence of PBS and human serum. Ex vivo biodistribution studies of [^68^Ga]Ga- MIONs showed high accumulation in the mononuclear phagocyte system (MPS) organs and satisfactory blood retention with time. In vivo PET imaging with [^68^Ga]Ga-MagP MIONs was in accordance with the ex vivo biodistribution results. Finally, the MIONs showed low toxicity against 4T1 breast cancer cells. These detailed studies established that [^68^Ga]Ga- MIONs exhibit potential for application as tracers for early cancer detection.

## 1. Introduction

In the pursuit of more effective cancer targeting probes, radiolabeled nanoparticles have attracted intense scientific interest for their great potential in nuclear medicine. The unique physical and chemical properties of materials in the nanoscale, such as high surface to volume ratio, magnetic properties, and tunable surface chemistry, have led to the development of numerous nanoparticle types of different size, shape, and core material as versatile platforms for various applications. Nanoparticles labeled with radioisotopes, such as Technetium-99m [^99m^Tc], Gallium-68 [^68^Ga], and Zirconium-89 [^89^Zr], are used as cancer imaging agents in single photon emission computed tomography (SPECT) and positron emission tomography (PET) [1,2,3,4,5,6,7,8]. For therapeutic purposes, nanoparticles are radiolabeled with beta (β)- or alpha (α)-emitting isotopes such as Lutetium-177 [^177^Lu] and Actinium-225 [^225^Ac], respectively [9,10,11]. Moreover, the conjugation of diagnostic and therapeutic radionuclides onto the same nanostructure provides theranostic capabilities [12,13]. Finally, the functionalization of these nanoparticles with various moieties transforms them into multimodal theranostic or therapeutic systems [14,15]. For example, radiolabeled particles that incorporate drugs can be used for image-guided drug delivery or controlled release with synergistic therapeutic effects (e.g., by combining radio- and chemotherapy) [16,17,18]. Magnetic iron oxide nanoparticles (MIONs) are among the most attractive theranostic nanoplatforms [19]. Apart from the advantages mentioned above, MIONs have exceptional characteristics that make them suitable for various uses in cancer management [20]. Firstly, due to their spin-active lattice and high biocompatibility, MIONs are clinically used as T2-contrast enhancement agents in MRI [21,22,23] and have great potential in magnetic force-guided drug delivery and cancer treatment with magnetic hyperthermia [24,25,26]. Secondly, the synthesis of MIONs by chemical procedures is facile and enables highly controllable size, composition, and magnetic properties [27]. Finally, radiolabeled MIONs can play the role of dual modality PET/MR or SPECT/MR imaging agents, combining the sensitivity of nuclear medicine modalities with the anatomical details provided by MRI, thus improving cancer diagnosis [15,27,28,29].

The delivery of MIONs to tumors is achieved mainly by active and passive targeting. For active targeting, the particle surface is functionalized with ligands (small molecules, peptides, monoclonal antibodies) that recognize specific molecular cancer targets [18,29,30,31]. Passive tumor targeting is based on the enhanced permeability and retention effect (EPR), which comprises the extravasation of nanoparticles through the intercellular pores of tumor vessels (leaky vasculature) and their retention in tumor interstitial space due to the abnormally poor lymphatic drainage [32,33]. Passive accumulation of MIONs in tumors is greatly determined by their retention time in blood circulation, which in turn can be controlled by particle size and surface chemistry tuning. Nanoparticles smaller than 10 nm are rapidly cleared from the bloodstream via the kidneys, while particles over 200 nm are quickly covered by plasma proteins, namely opsonins; recognized by monocytes and macrophages; and finally undergo phagocytosis in the mononuclear phagocyte system (MPS) organs (mainly liver, spleen, and bone marrow). Hence, nanoparticle size between 10 and 200 nm is mostly suitable for a long circulation time, which increases the possibility for effective tumor uptake [27,34,35]. With respect to surface modification, the presence of neutral and hydrophilic polymeric coatings (e.g., PEG, dextran, and silica) has been correlated with reduced nonspecific interactions and thus stealthy delivery [36,37]. Moreover, suitable coating imparts colloidal stability (agglomeration avoidance), low toxicity, and protection of the magnetic properties of the MIONs, necessary characteristics for a cancer targeting candidate [36].

Early cancer diagnosis remains a key factor for curative treatment and increased survival [38]. PET, based on the detection of coincidence photons produced by positron-electron annihilation, is the most sensitive imaging technique able to quantify the acquired information, thus offering early detection, which leads to improved decision making [39]. The choice of the radioisotope is a crucial parameter when considering the design of an effective imaging agent. While ^18^F is the most common PET isotope in the clinic, ^68^Ga has been at the center of scientific studies for the last 20 years due to its favorable characteristics. ^68^Ga mainly decays by positron emission (88.8%) to stable ^68^Zn, with a half-life of 67.83 min. The high emission probability of annihilation photons results in a strong signal for detection with PET systems, while the relatively low yield of other γ-photons and short half-life are positive features from the radiation protection point of view. Another advantage of ^68^Ga is its easy onsite availability from a ^68^Ge/^68^Ga generator (T_1/2_ of ^68^Ge = 270.8 days); hence, there is no need of a locally or nearby-installed cyclotron for everyday production. Lastly, thanks to its versatile coordination chemistry, ^68^Ga(III)—the prevalent state in aqueous solutions—conjugates with a variety of ligands that contain functional groups, such as carboxylates, phosphonates, and amines, to form thermodynamically stable complexes with various agents for imaging applications [2]. All the above were the driving force of a plethora of studies concerning ^68^Ga-labeled nanoparticles, the results of which are promising for new ^68^Ga-based cancer imaging probes in the future [3,5,6,7].

In this study, two different approaches were explored to develop MIONs radiolabeled with ^68^Ga. The MIONs were initially functionalized with the poly(methacrylic acid)-graft-poly(ethyleneglycol methacrylate) copolymer (p(MAA-g-EGMA)). The primary idea behind the design and synthesis of these MNPs was to improve stability and biocompatibility, as well as to provide a platform for the development of a theranostic agent. Then, the chelating agent NODAGA-NHS was conjugated onto the surface of the MIONs (MagP-NODAGA) to form a chelate complex with ^68^Ga in the chelator-mediated approach. In the chelator-free approach, ^68^Ga was directly incorporated onto the surface of the MIONs (MagP) by taking advantage of its affinity towards the carboxylic groups of the copolymer coating. In vitro stability studies were performed with [^68^Ga]Ga-MIONs to assess the stability of the NPs in phosphate-buffered saline (PBS) and human serum, while in vitro cytotoxicity studies of the PEGylated MIONs were performed to evaluate their cytotoxicity. To examine the potential of the [^68^Ga]Ga-MIONs as radiotracers for early cancer detection, biodistribution studies were conducted in normal mice, while initial micro-PET imaging studies were also performed on an experimental tumor model.

## 2. Materials and Methods

^68^Ga is a high-energy positron emitter, which presents serious health threats and requires special radioprotective precautions during handling to reduce the risk of harm. All radiolabeling procedures and work associated with radiolabeled compounds was conducted in a radiochemistry facility which has all the necessary infrastructure, expertise, and licensing to safely conduct experiments with radioisotopes.

Materials used for the synthesis of the MIONs were purchased from Lab NV (FeSO_4_ × 7H_2_O), Carlo Erba (37% HCl), Aldrich (tris(2aminoethyl)amine and N,Nʹ-diisopropylcarbodiimide), Acros Organics (DMF), and CBL Patras (hydroxybenzotriazole). The chelating agent NODAGA-NHS was acquired from CheMatech (Dijon, France). Ultrapure 3D-H_2_O with conductivity ~1 μS/cm from an ELGA MEDICA apparatus was used for all the experiments. Additionally, NaOH and NaCl were from Merck chemicals and NH_4_OH (30%, for analysis) from Carlo Erba (Sabadell, Barcelona)). It is noted that all other chemicals not specified were supplied from Sigma-Aldrich (Darmstad, Germany).

The buffer used for radiolabeling was prepared from trace-free reagents (Sigma-Aldrich). The 4T1 breast cancer cell line was acquired from the cell bank of the Laboratory of Radiobiology, Institute of Nuclear & Radiological Sciences and Technology, Energy & Safety, NCSR “Demokritos”. Cells were free of mycoplasma contamination, as judged visually under microscope observation and by regular 4′,6-diamidine-2′-phenylindole dihydrochloride (DAPI) staining of the cell cultures. The media for the cultures were purchased from Biowest (Riverside, MO, USA), and the MTT reagent (3-[4,5-dimethylthiazol-2-yl]-2,5-diphenyl-tetrazolium bromide) was obtained from Applichem (Darmstad, Germany). Optical density measurements in in vitro experiments were conducted using a LabSystems Multiskan RC Microplate Reader (Thermo Fisher ScientificWaltham, MA, USA). A lower-activity commercial Ge-68/Ga-68 generator was acquired from Eckert & Ziegler (Berlin, Germany). Water for injection was purchased from DEMO S.A. All other reagents and solvents used in these studies were obtained from commercial sources without further purification. Radioactivity of the [^68^Ga]GaCl_3_ and the radiolabeled nanoparticles was measured using a dose calibrator (Capintec, Ramsey, NJ, USA). Whatman Grade 1 chromatography paper was purchased from Sigma-Aldrich and, along with a Radio-TLC Scanner (Scan-Ram, LabLogic, Sheffield, UK), was used in the determination of radiolabeling yield/purity and in vitro stability studies. Water was deionized to 18 MΩ⋅cm using an Easypure water filtration system (Barnstead International, Dubuque, Iowa). A gamma scintillation counter, Cobra II, Canberra, Packard, was used to measure the radioactivity of each of the organ and blood samples in ex vivo biodistribution studies.

Animals used for the biodistribution studies were obtained from the breeding facilities of the Institute of Biosciences and Applications, NCSR “Demokritos”. Our experimental animal facility is registered according to the Greek Presidential Decree 56/2013 (Reg. Number: EL 25 BIO 022), in accordance with the European Directive 2010/63, which is harmonized with national legislation, on the protection of animals used for scientific purposes. All applicable national guidelines for the care and use of animals were followed. The study protocol was approved by the Department of Agriculture and Veterinary Service of the Prefecture of Athens (Protocol Number: 1607/11-04-2018). These studies have been further approved by our institutional ethics committee, and the procedures followed are in accordance with institutional guidelines.

PET scans were performed using a Mediso scanner NanoScan PC, with 8 detector modules. This type of scanner has an axial field of view of 98.6 mm and a spatial resolution of 0.8 mm at the center of the scanner, employing the Tera-Tomo 3D PET iterative reconstruction algorithm. Prior to PET imaging, a CT scan was performed for attenuation correction and anatomic localization. The X-ray beam energy was set at 50 kVp, the exposure time at 300 msec, and current at 670 μA. The number of projections per rotation was 480, and the slice thickness was 250 μm. PET and CT data were reconstructed using Nucline software version 3.00.021.0000. The PET image reconstruction was performed using a version of 3D OSEM algorithm (Tera-Tomo 3D PET image reconstruction algorithm) with voxel dimensions of 0.4 × 0.4 × 0.4 mm (size of image matrix 83 × 98 × 392). Dead time, decay, scatter, attenuation, and axial sensitivity corrections and normalization were applied to PET data. Image analysis was performed with the Mediso InterView Fusion software package version 3.00.039.0000 BETA.

### 2.1. Synthesis of MIONs

#### 2.1.1. Synthesis and Functionalization of Magnetic Nanoparticles

The magnetic nanoparticles were synthesized following hydrolytic alkaline precipitation from a ferrous salt precursor route in the presence of p(MAA-g-EGMA) copolymer as previously described, with slight modifications [40]. In brief, FeSO_4_ × 7H_2_O (1.44 g, 99+%) was dissolved in 20 mL of deionized H_2_O (containing 60 μL of 37% HCl). The copolymers of poly(methacrylic acid)-graft-poly(ethyleneglycol methacrylate) were synthesized by radical copolymerization of methoxy-PEG-methacrylate with methacrylic acid, as previously described [41]. P(MAA-g-EGMA) (300 mg) was dissolved in dH_2_O (60 mL) and 4 mL NH_3_ (30%) was added to the polymer solution. The mixture was heated at 50 °C under magnetic stirring and the reaction stopped after 1 h and 30 min. The product was purified from byproducts by centrifugation at 18,000 rcf for 30 min and subsequent redispersion of the pellet in dH_2_O, followed by sonication and vortexing. The procedure was repeated thrice. A final mild centrifugation (2100 rcf, 15 min) was performed for the fractionation of the product (MagP), in order to remove larger aggregates.

Functionalization of MagP with tris(2aminoethyl)amine (96%) was performed according to the following protocol. The acid form of MagP was prepared by dialysis in H_2_O at pH 4 for 3 days, exchanging the bath medium frequently. The MagP nanoparticles were then transferred in DMF by centrifugation and subsequent redispersion of the pellet in DMF. The process was repeated thrice. DIC (N,Nʹ-diisopropylcarbodiimide) and HOBt (hydroxybenzotriazole) coupling reagents were used for the conjugation of tris(2aminoethyl)amine onto the carboxylates of the nanoparticles. Conjugation reagents were used in a molar excess of 3.3 with respect to the carboxylates present on MagP, while triamine was used in a molar excess of 3. Finally, the amine-functionalized nanoparticles were mixed with the amine-reactive chelating agent NODAGA-NHS (ester of a macrocyclic chelator NODA derivative with N-hydroxysuccinimide) and left for overnight magnetic stirring, resulting in the chelator-modified MagP-NODAGA nanoparticles. After each coupling reaction, nanoparticles were thoroughly washed through centrifugation and redispersion in DMF. The final product was redispersed in distilled H_2_O and dialyzed for several days with dialysis membranes in order to remove DMF traces and reaction byproducts.

#### 2.1.2. Hydrodynamic Diameter and Zeta Potential Measurements

Dynamic light scattering (DLS) was performed on aqueous dispersions of ∼0.01% *w*/*v* in Fe_2_O_3_, where scattered light was collected at a fixed angle of 173° with a Malvern Instrument ZetaSizer Nano and with a 4-mW He−Ne laser, operating at a wavelength of 633 nm. The hydrodynamic diameters (*D*_h_) and the reported polydispersity index (PDI) values are the mean of three measurements, where each measurement was the sum of 12 correlograms and fitting procedures. The cumulants analysis method was applied, and the *D*_h_ average values reported are the z-average mean *D*_h_, unless otherwise stated. Electrokinetic measurements for the determination of the mobility and zeta-potential (ζ-p) values of the suspensions were performed using the same instrument as the average of 100 runs with the phase analysis light scattering mode, after equilibration at 25 °C.

#### 2.1.3. Thermogravimetric Analysis (TGA)

The polymer content in the hybrid materials was determined with thermogravimetric measurements (TA Instruments, Q500). Measurements were performed under N_2_ flow at a heating rate of 10 °C/min from room temperature to 700 °C.

#### 2.1.4. Transmission Electron Microscopy (TEM) 

Samples for TEM were prepared by casting a droplet of a dilute aqueous suspension (0.01% *w*/*v* in Fe_2_O_3_) of the hybrids on copper grids coated by Formvar carbon film. Micrographs were obtained by a JEOL, JEM-2100 instrument operating at 200 kV.

#### 2.1.5. Magnetophoretic Evaluation

The magnetophoretic experiments were performed using a Hitachi Digilab U-2800 spectrophotometer, by inserting a cylindrical Nd−Fe−B magnet (diameter = 20 mm, thickness = 10 mm) next to the cuvette holder at a distance of 2 mm, as previously described [40]. The absorbance of the magnetic nanoparticles was recorded every 100 s for 7000 s at 450 nm. The concentration of all the magnetic colloids in all experiments was set at 0.012% *w*/*v* Fe_2_O_3_.

### 2.2. Cell Cultures

4T1 cells (murine mammary carcinoma) were grown in RPMI-1640 Medium (RPMI) of pH 7.0 to 7.4, supplemented with fetal bovine serum (FBS) to a final concentration of 10%, 100 U/mL of penicillin, 2 mM glutamine, and 100 μg/mL of streptomycin. Cell cultures were maintained in flasks and were grown at 37 °C in a humidified atmosphere of 5% CO_2_ in air. Subconfluent cells were detached using a 0.25% trypsin-0.53 mM ethylenediaminetetraacetic acid (EDTA) solution, and the subcultivation ratio was 1:8–1:10.

### 2.3. MTT Toxicity Assay

In vitro cytotoxicity of the prepared MagP and MagP-NODAGA MIONs against 4Τ1 cells was evaluated by the MTT colorimetric assay. MTT (3-(4,5-dimethylthiazol-2-yl)-2,5-diphenyltetrazolium bromide) is a yellow tetrazolium salt that is reduced by metabolically active cells in mitochondria, via the action of dehydrogenase enzymes. The resulting product, a purple formazan, can be solubilized and quantitatively determined by spectrophotometry. Absorbance is proportional to the concentration of formazan, thus to the concentration of active cells. The inability of dead cells to metabolize MTT leads to reduced absorbance, which subsequently can be used as an indicator of cytotoxicity. For the toxicity assay, cells were seeded in 96-well plates (15 × 10^3^ cells/well) and allowed to grow overnight at 37 °C in a 5% CO_2_ incubator. Cells were incubated with increasing concentrations of both complexes (1.0625, 3.125, 6.25, 12.5, 25, and 50 μg (Fe_2_O_3_)/mL) for 24 h [42]. The medium was then removed and replaced with 100μL of MTT dissolved in RPMI (1 mg/mL). After a 4-h incubation period, the MTT solution was carefully aspirated, and formazan crystals were solubilized with 100μL of isopropanol. Absorbance was recorded at 540 nm. All experiments were performed in triplicate, and the results were calculated as noted from the following equation: cell viability (%) = (mean optical density (OD) in treatment group/mean OD in control group) × 100.

### 2.4. Labeling of MIONs with ^68^Ga and Radiochemical Analysis

For the labeling of the magnetic nanoparticles, ^68^Ga was initially eluted from a ^68^Ge/^68^Ga generator with 7 mL 0.1 N HCl and trapped onto an acidic cation-exchange resin. Metal impurities were removed by passage of 1 mL of a solution containing acetone (80 *v*/*v*%) and 0.15 M HCl (20 *v*/*v*%). Desorption of purified ^68^Ga was accomplished with 400 μL of a solution of acetone (97.6 *v*/*v*%) and 0.15 M HCl (2.4 *v*/*v*%) [43]. Then, a mixture containing 350 μL of 0.5 M sodium acetate buffer pH 4, 50 μL of MIONs suspension (C_MagP_ = 2.8 mg/mL, C_MagP-NODAGA_ = 1.1 mg/mL), and 100 μL of [^68^Ga]GaCl_3_ (~40 MBq) was vortexed and incubated at 70 °C for 30–120 min. The activities of the eluate and labeling mixture were measured with a dose calibrator. The radiochemical yield (RCY) of the labeled MIONs was determined at three time points after incubation (30, 60, and 120 min) by thin layer chromatography, using Whatman No 1 sheets as the stationary phase and 0.1 M citric acid as the mobile phase. In this chromatography system, the [^68^Ga]Ga-MIONs remain at the application point, while unbound ^68^Ga ions move to the solvent front [3]. The latter was verified by a control experiment, where the MIONs suspension was replaced by an equal volume of ultrapure water, while all the other constituents of the sample remained the same. The developed sheet was scanned by a radio-TLC detector and the %RCY of [^68^Ga]Ga-MIONs was calculated as: 100 × (counts at application point/total counts). Data collection and analysis were performed with Laura software v. 5.0.4.29 (Sheffield, UK).

### 2.5. In Vitro Stability Studies 

In vitro stability of the [^68^Ga]Ga-MIONs was tested in phosphate-buffered saline (PBS) and human serum. For this purpose, 20 μL of radiolabeled nanoparticles were incubated with 180 μL PBS pH 7.4, while shaking at room temperature. The serum stability studies were conducted using human serum (Sigma–Aldrich) according to the procedure followed with PBS, except for the incubation temperature (37 °C). The stability was evaluated at 30, 60, and 120 min by radio-TLC (as described above) and the experiments were performed in triplicate.

### 2.6. Animal Models

For the animal experiments, female normal Swiss albino and athymic SCID mice were used. The animals were housed in air-conditioned rooms under a 12-h light/dark cycle and allowed free access to food and water. For the development of experimental tumors, 1 × 10^6^ 4T1 mouse mammary carcinoma cells were subcutaneously inoculated into the front left limb of SCID mice. Studies were carried out on the 6th day after cell inoculation.

### 2.7. Ex Vivo Biodistribution Studies

The biological behavior of [^68^Ga]Ga-MagP and [^68^Ga]Ga-MagP-NODAGA MIONs was evaluated in normal Swiss mice (20–30 g) as well as in SCID mice (15–25 g) bearing 4T1 tumors (*n* = 3–5 animals per time point). According to the experimental protocol, 100μL/~300 kBq of diluted radiolabeled MIONs suspension (containing 1.4 μg of [^68^Ga[Ga-MagP/0.55 μg [^68^Ga]Ga-MagP-NODAGA MIONs) (dilution 1:20 in water for injection) was counted in a dose calibrator and administered to the animals via the tail vein. At 30, 60, and 120 min postinjection, the animals were euthanized, and the organs and tissues of interest (including the tumor, in the case of the tumor model) were collected, weighed and measured in an automatic gamma counter. For the calculation of the injected dose in each animal, the radioactivity remaining in the tail was subtracted, and a standard solution was used. All measurements were corrected for background and radioactive decay. Finally, the accumulation of the radiolabeled MIONs in organs and tissues at each time point was expressed as the mean percentage of injected dose per gram ± standard deviation (% ID/g ± SD).

### 2.8. In Vivo Imaging Studies (Pilot Studies)

In order to further assess the passive accumulation of [^68^Ga]Ga-MIONs in tumor-bearing mice, in vivo micro-PET scans were performed on a 4T1 tumor model. Tumor-bearing mice were fasted for a period of 12 h prior to imaging. The PET studies were conducted using 2% isoflurane anesthesia in oxygen at 0.8 lt/min. The lateral tail vein of the mouse was injected with 100μL/7.42MBq of [^68^Ga]Ga-MagP. Radiotracer uptake time was 120 min p.i. and was followed by a 20-min PET static scan. During uptake time, the animal was anesthetized, and its body temperature was maintained at 36 °C on the scanner bed.

### 2.9. Statistical Analysis

The data are presented as means ± standard deviations (SD). For the in vitro stability and biodistribution studies, the data were compared using an unpaired *t*-test with a significance level of *p* < 0.05. Asterisks indicate the statistical significance of the difference between the results (* *p* < 0.05, ** *p*< 0.01, *** *p* < 0.001). Absence of asterisks denotes a nonsignificant statistical difference. Analyses were performed using GraphPad Prism (San Diego, CA, USA) and Matlab 9.2 (R2017a) (Santa Rosa, CA, USA) software.

## 3. Results and Discussion

### 3.1. Synthesis, Functionalization, and Magnetophoretic Evaluation of MIONs

**Figure 1:**
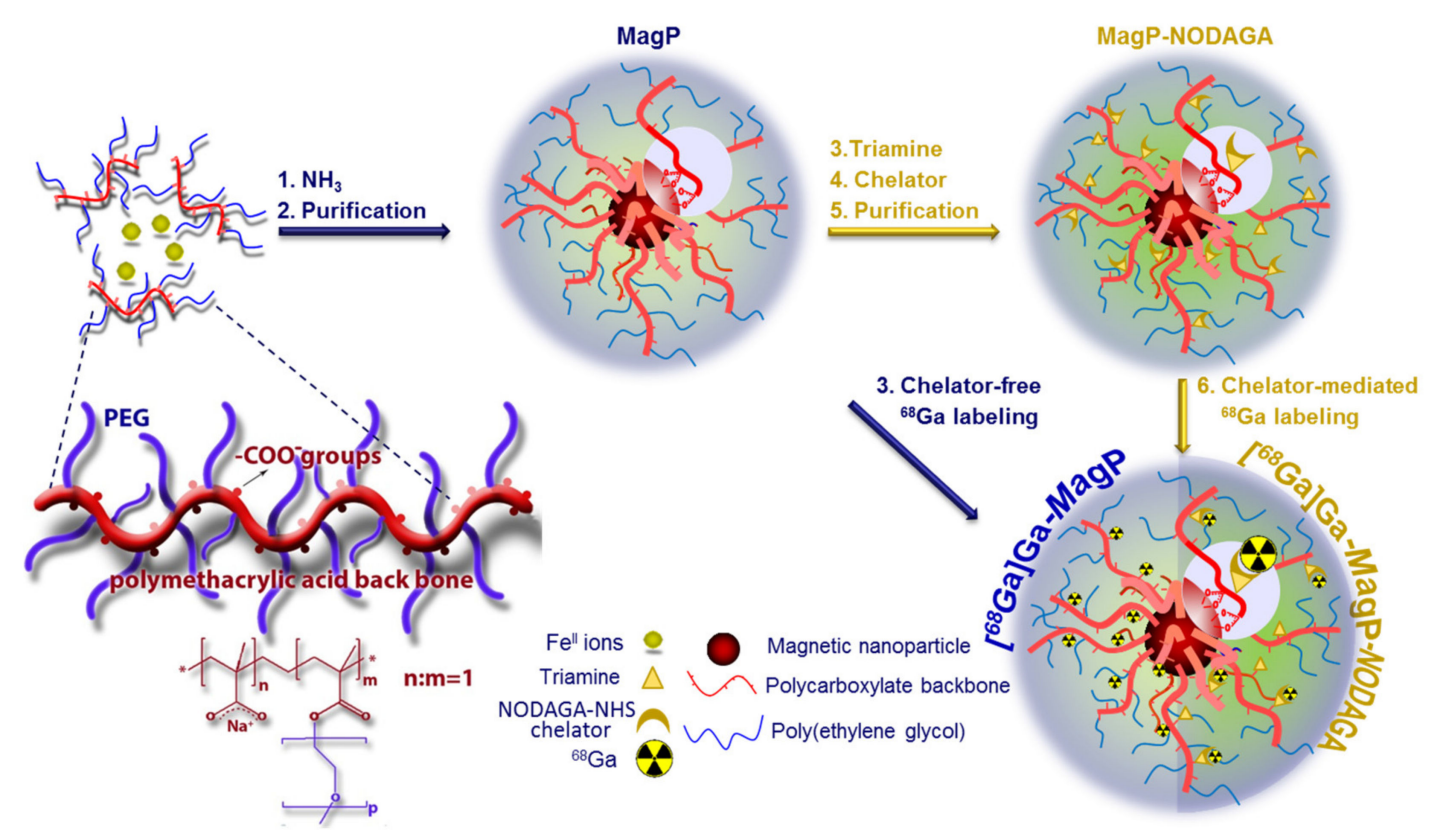
The steps followed for the synthesis of MagP MIONs and the radiolabeled derivatives through chelator-free or chelator-mediated radiolabeling. The structure of the PEG-branched copolymer of poly(methacrylic acid)-graft-poly(ethyleneglycol methacrylate) is also shown.

The synthesis of the hybrid MIONs was realized by hydrolytic condensation of a single ferrous precursor in the presence of the p(MAA-g-EGMA) copolymer acting as the surface functionalizing agent through a simple self-assembly process. The carboxylate groups of the methacrylic monomers coordinate with the Fe ions of the growing MIONs, while the PEG polymer chains mainly populate the outer corona, forming colloidal clusters, as previously observed [40,44]. The p(MAA-g-EGMA) copolymer has a high content of anionic carboxylic sites, facilitating the effective functionalization with the chelating agent for ^68^Ga (Figure 1).

**Figure 2:**
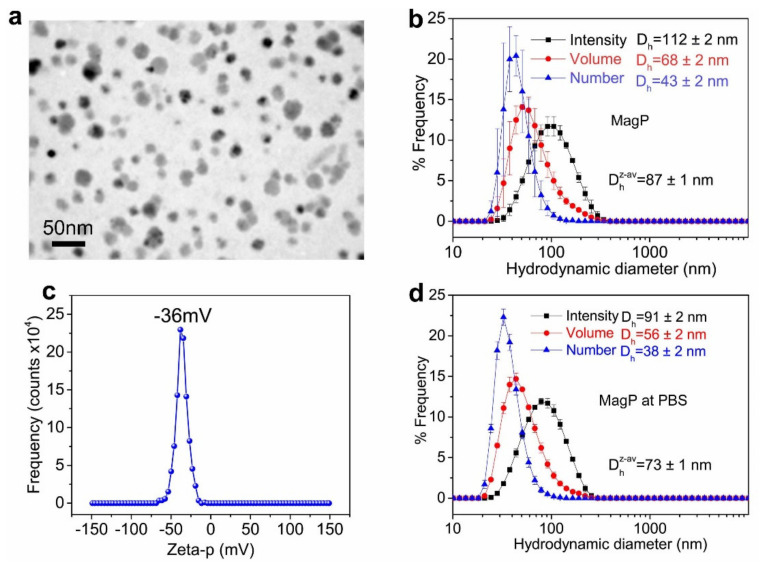
(**a**) TEM image; (**b**) hydrodynamic diameter distributions from DLS measurements; (**c**) zeta-potential distribution for the MagP nanoparticles; (**d**) hydrodynamic diameter distributions from DLS measurements for the MagP-NODAGA functionalized MIONs. The lower D_h_ of MIONs in PBS is attributed to the contraction of the polyelectrolyte chains due to the high salinity.

**Figure 3:**
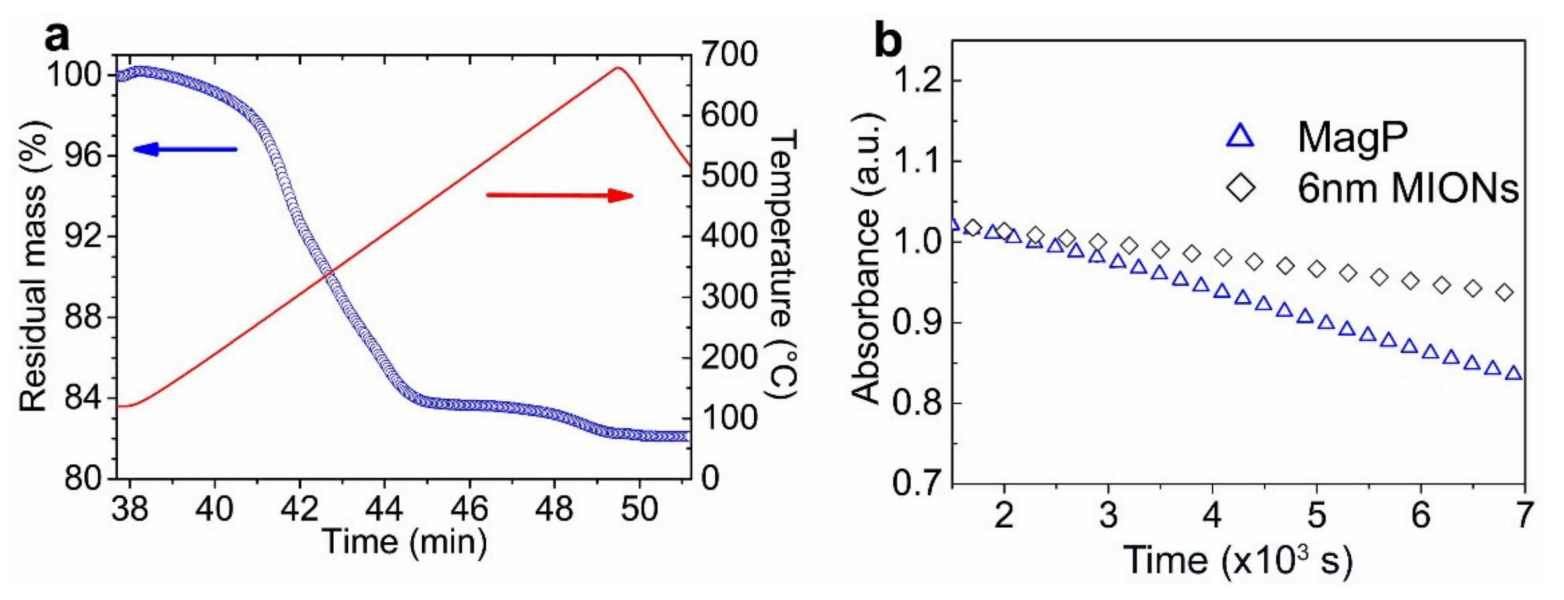
(**a**) Thermogravimetric analysis for the MagP MIONs; (**b**) magnetophoretic response of the MagP MIONs compared to samples composed of small magnetic nanocrystals with low Ms (~35 emu/g).

TEM images (Figure 2a) confirmed the growth of nanoparticles with multiple nanocrystallites forming small clusters of 30–40 nm in diameter due to polymer bridging. The synthesized MagP MIONs displayed an average D_h_ of 87 nm (Figure 2b), which is suitable for passive targeting of cancerous tissues through the EPR effect after intravenous administration [45]. Electrokinetic measurements showed a negative ζ-p of −36 mV at physiological pH (Figure 2c), due to the presence of the negatively charged –COO− groups of the polymer. The negative ζ-p, along with the PEG chains of the polymer coating, provides a combination of strong electrosteric and steric colloidal stability. This was further corroborated by stability assays in PBS, where MIONs perfectly retained their hydrodynamic diameter after incubation for several hours in PBS (Figure 2d). This characteristic indicates effective steric stabilization, which can prolong the blood circulation time of the nanoassemblies when administered in vivo. The polymer content of the MagP nanoparticles was estimated through thermogravimetric analysis (TGA, Figure 3a) under a N_2_ stream at 18 wt.%. Thus, the particularly high 82 wt.% magnetic content in the MagP MIONs ascribes strong magnetic properties to the colloids. Magnetophoretic experiments demonstrated the effective manipulation of nanoparticles through the application of external magnetic gradients. The freshly synthesized MagP MIONs displayed almost identical behavior compared to previously reported synthesized batches with similar physicochemical characteristics (D_h_ = 85 nm) and a high saturation magnetization (Ms) of 80 emu/g [40,46]. For comparison, a sample composed of 6-nm MIONs, and thus of significantly lower magnetization (~35 emu/g), is also included in Figure 3b to demonstrate the difference in magnetic response.

**Figure 4:**
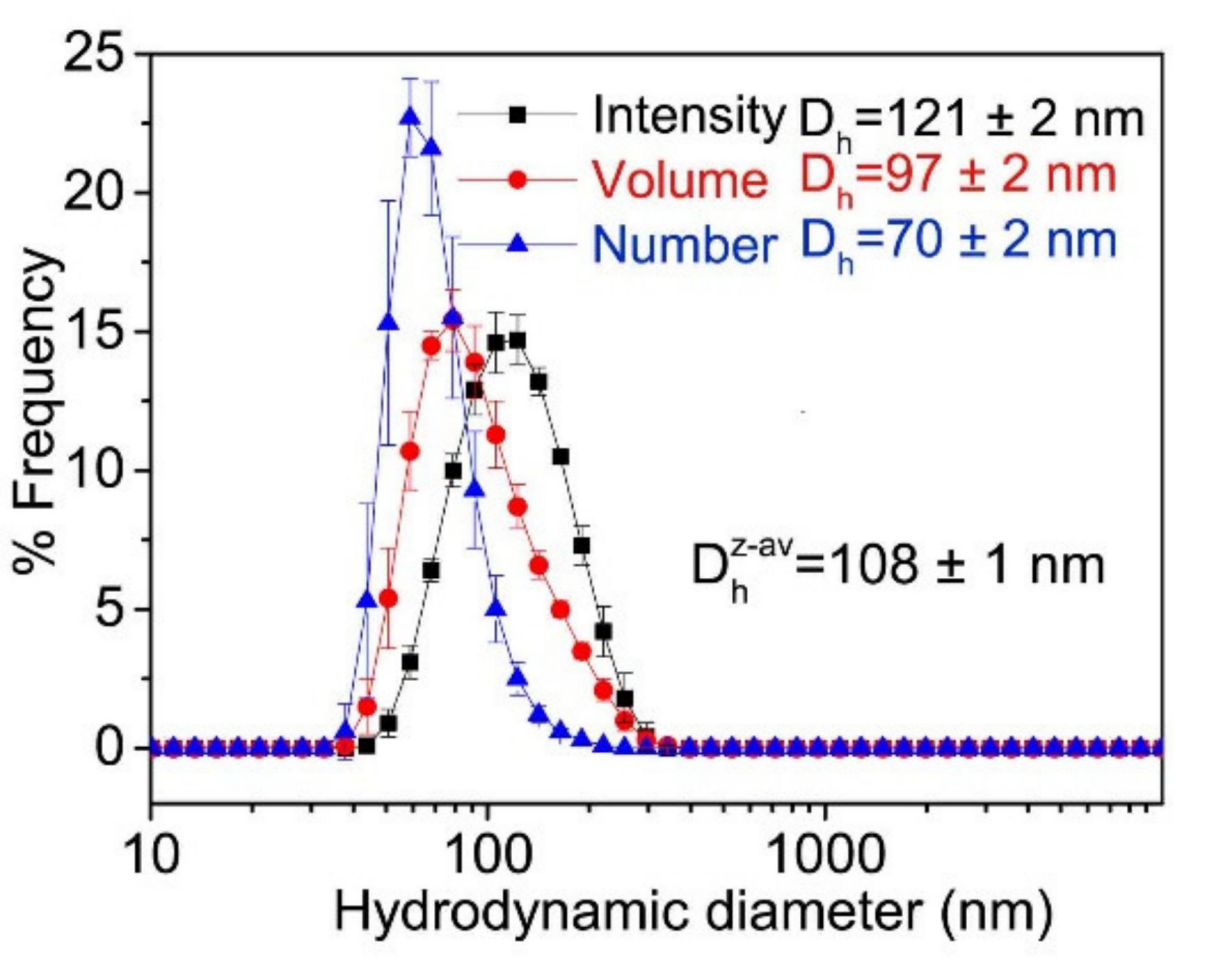
Hydrodynamic diameter distributions from DLS measurements for the MagP-NODAGA MIONs.

The abundant carboxylic groups of the MagP MIONs were utilized for the modification of nanoparticles with a triamine. This step is required for the subsequent conjugation with the amine-reactive chelator NODAGA-NHS ester. The resulting MagP-NODAGA MIONs exhibited a slightly larger D_h_ of ~108 nm (Figure 4).

### 3.2. Cytotoxicity of MagP and MagP-NODAGA MIONs

**Figure 5:**
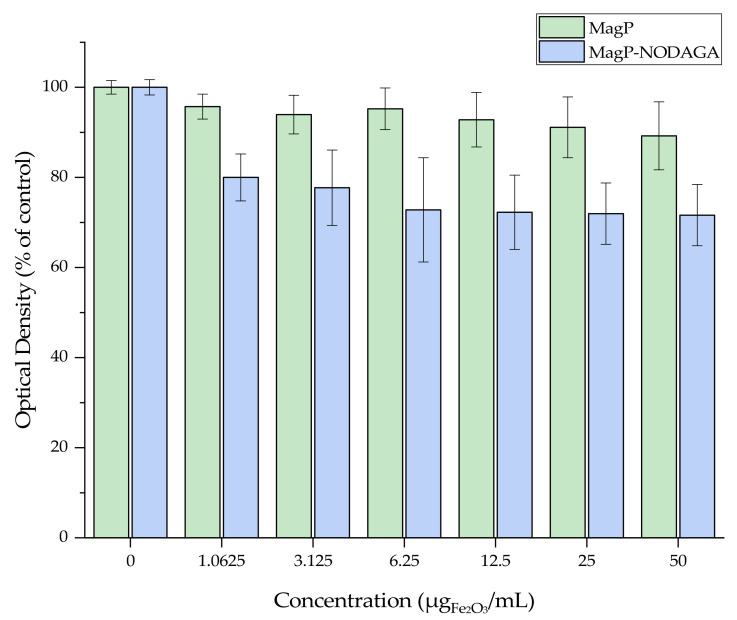
MTT assay of 4T1 cells treated with different concentrations of MagP and MagP-NODAGA MIONs. Zero concentration corresponds to the negative control for both NPs (no nanoparticles present in the sample). Mean values (*n* = 3) and the SD (bars) are shown.

The MTT assay was conducted to assess the cytotoxicity of the MagP and MagP-NODAGA MIONs against 4T1 breast cancer cells. Figure 5 summarizes the experimental data after 24 h of cell exposure to different concentrations of the nanoparticles. MagP were not cytotoxic, and even at the highest tested concentration of 50 μg (Fe_2_O_3_)//mL, a viability of about 90% was observed. However, in the case of MagP-NODAGA, cell viability values tended to decrease gradually from 80% for the lower concentration of 1.0625 μg (Fe_2_O_3_)//mL) to 71% at the highest concentration of 50 μg (Fe_2_O_3_)/mL. The slightly higher toxicity of MagP-NODAGA compared to MagP MIONs could possibly be attributed to the presence of the NODAGA-NHS chelating agent in the former species.

### 3.3. Labeling of MIONs with ^68^Ga

The labeling procedure of the magnetic nanoparticles consisted of the incubation of a mixture of [^68^Ga]GaCl_3_ eluate and nanoparticles at 70 °C, pH 4 (as described above). These conditions were finalized after preliminary experiments were conducted, taking into account the existing literature on gallium chemistry. In aqueous solutions, gallium is primarily found in the oxidative state +3. Ga(III), as a hard Lewis acid, binds to electron donors, such as oxygen and nitrogen. However, bonding is not favored by extreme pH, since in a strongly acidic environment, the protonation of donor atoms occurs, and insoluble gallium hydroxides are formed at high pH values. Thus, the pH range 3–5 is considered as most suitable for labeling with gallium [2,3]. In our study, MagP-NODAGA MIONs were labeled by chelate complex formation of ^68^Ga(III) with NODAGA-NHS through the 3 nitrogen atoms of the macrocyclic ring and the 3 oxygen atoms of the carboxylate groups of the chelator [47]. On the contrary, the labeling of MagP MIONs was direct, through the coordination of the radioisotope with the carboxylates of the copolymer p(MAA-g-EGMA).

**Figure 6:**
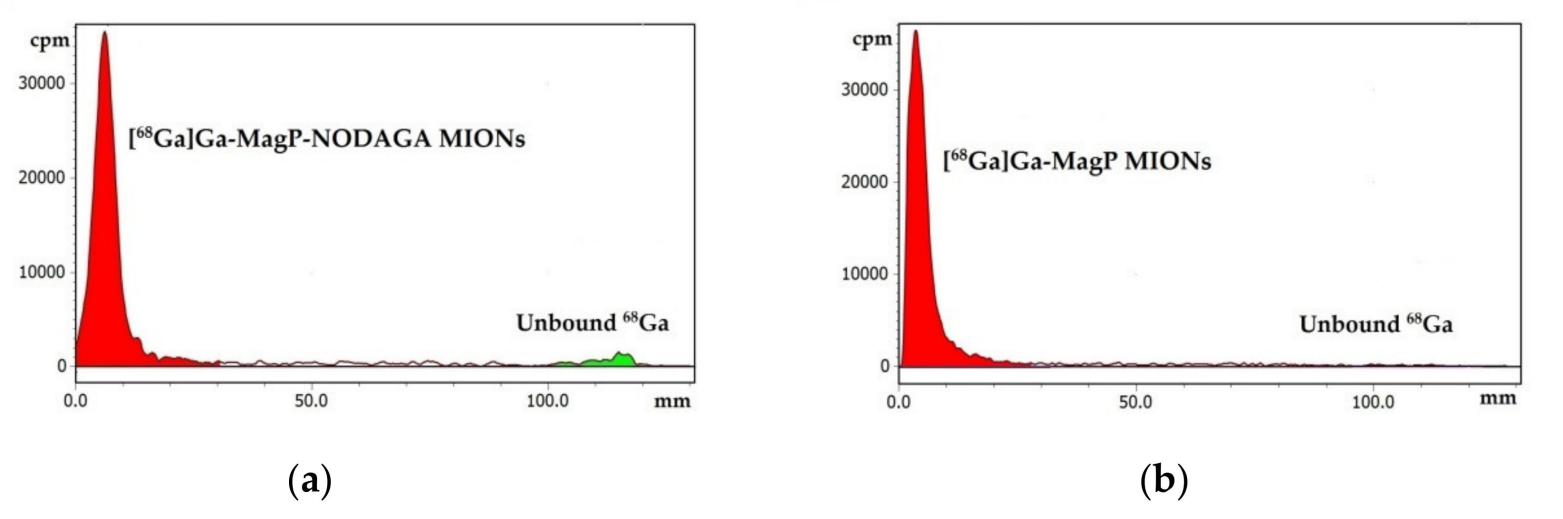
Radio-TLC chromatograms of ^68^Ga labeling of: (**a**) MagP-NODAGA and (**b**) MagP MIONs. The peak at the spot area (first 30 mm) is attributed to the radiolabeled MIONs, whereas at the front, a small amount of unbound ^68^Ga is detected. These results are acquired after 30 min of incubation.

**Figure 7:**
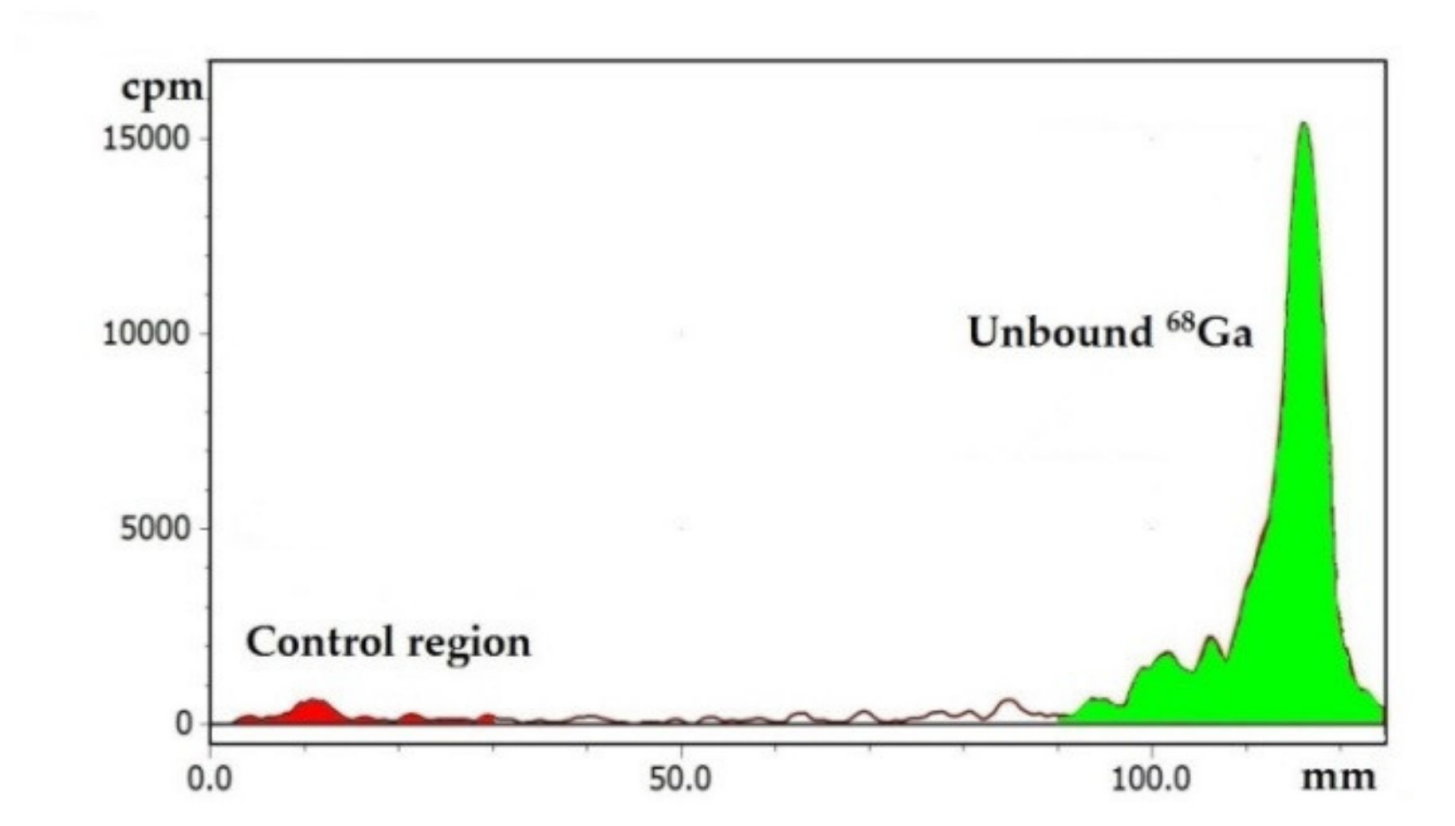
Radio-TLC chromatogram of a control experiment (30 min incubation). In the absence of nanoparticles, free ^68^Ga ions move to the front; thus, the highest amount of radioactivity is detected in this area.

According to the radio-TLC analysis results, the radiochemical yields were > 92% for the MagP-NODAGA MIONs and > 95% for the MagP MIONs after 30 min of incubation (Figure 6). Doubling the reaction time resulted in no significant improvement. Control experiments, conducted using ultrapure water instead of nanoparticle suspension, confirmed the efficacy of the labeling, as > 96% of the radioactivity was detected at the solvent front, where unbound ^68^Ga ions migrate (Figure 7).

Overall, the labeling protocol followed resulted in highly efficient radiolabeling of both groups of magnetic nanoparticles with ^68^Ga. Labeling was achieved rapidly (in 30 min), which is very important, as fast reactions are required taking into account the short half-life of ^68^Ga. Moreover, the conjugation of ^68^Ga to MagP MIONs indicates that the macrocyclic chelator NODAGA-NHS is not necessary for the successful radiolabeling of these nanostructures.

### 3.4. In Vitro Stability of [^68^Ga]Ga-MIONs

Stable coordination of ^68^Ga to the nanoparticles, for an adequate time after their administration, is important for their effectiveness as imaging agents. In vivo, free ^68^Ga binds to plasma proteins and, generally, is subject to nonspecific interactions [4]. Thus, under conjugate instability, the acquired PET images depict the biodistribution of the unbound radioisotope and not the areas of nanoparticle accumulation.

In vitro stability of radiolabeled nanoparticles is indicative of their corresponding in vivo behavior. For this reason, before proceeding to animal experiments, the stability of [^68^Ga]Ga-MIONs was assessed in PBS and human serum at 3 time points, up to 120 min, in an attempt to simulate physiological conditions.

**Figure 8:**
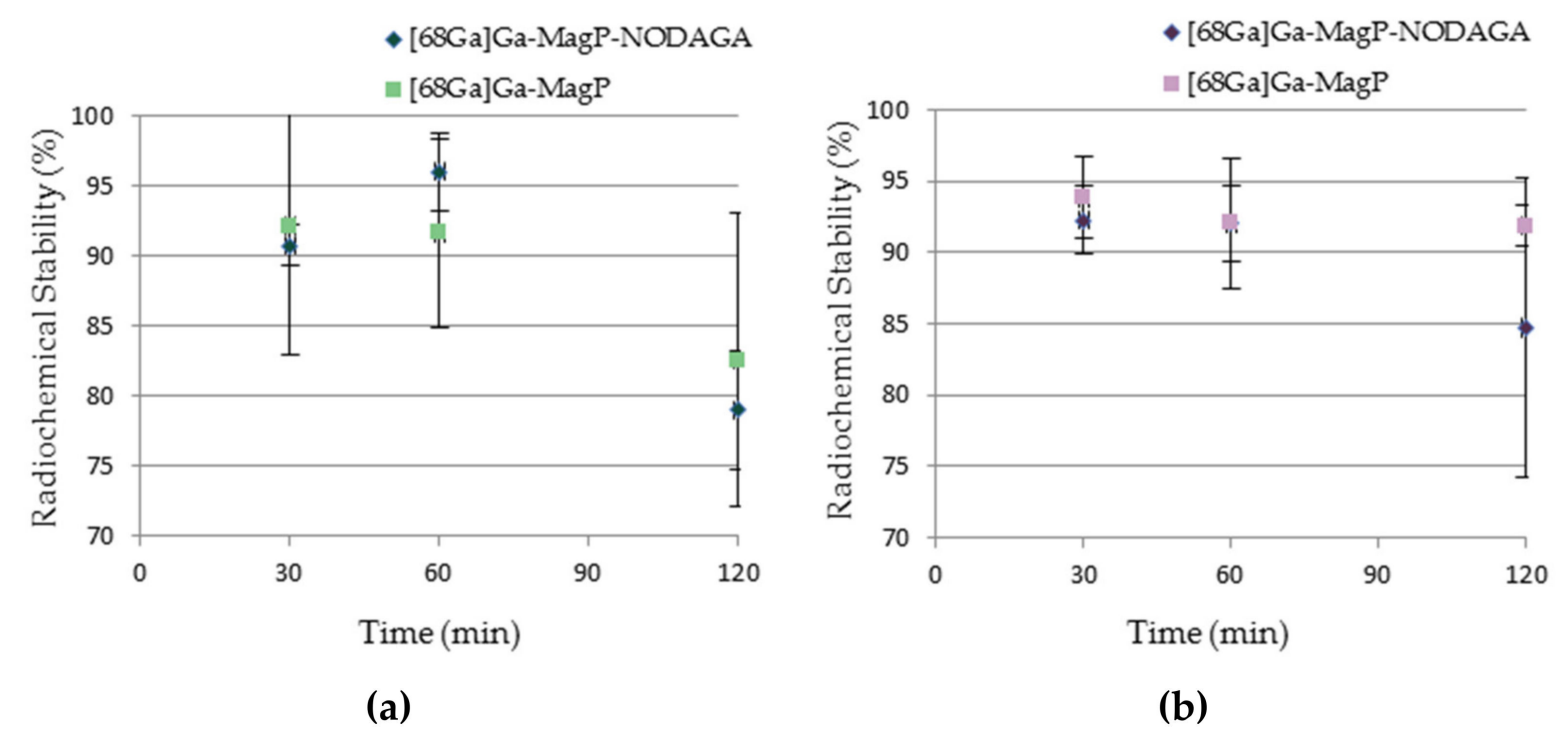
Radiochemical stability of [^68^Ga]Ga-MIONs in: (**a**) PBS and (**b**) human serum. A statistical difference was found between stability results of [^68^Ga]Ga-MagP-NODAGA MIONs in PBS at 60 and 120 min (*p* < 0.01) and 30 and 120 min (*p* < 0.05).

After radio-TCL analysis, it was found that 78.99 ± 4.27% of [^68^Ga]Ga-MagP-NODAGA MIONs remained intact after 2 h of incubation in PBS, while the respective percentage for [^68^Ga]Ga-MagP MIONs was 82.59 ± 10.48%. In human serum, the stability of [^68^Ga]Ga-MagP-NODAGA and [^68^Ga]Ga-MagP MIONs was 84.54 ± 10.53% and 91.86 ± 1.46%, respectively, at 120 min. It is noted that for both nanostructures, the stability results between time points were not statistically different, with the exception of [^68^Ga]Ga-MagP-NODAGA in PBS (Figure 8).

The satisfactorily high stability of both [^68^Ga]Ga-MIONs, especially in human serum, which is in accordance to similar studies reported in the literature [5,6,7], allowed us to proceed to biodistribution studies in mice models.

### 3.5. Ex Vivo Biodistribution Studies of [^68^Ga]Ga-MIONs

In order to evaluate the in vivo kinetics of [^68^Ga]Ga-MIONs, ex vivo biodistribution studies were performed in normal and 4T1 tumor-bearing SCID mice. The aim of these studies in normal mice is the assessment of the pattern of distribution of the MIONs in organs and tissues, as well as their excretion route. Biodistribution experiments in tumor-bearing mice were conducted in order to investigate the accumulation potential of the developed [^68^Ga]Ga-MIONs in solid tumors through passive targeting.

#### 3.5.1. Ex Vivo Biodistribution Studies of [^68^Ga]Ga-MIONs in Normal Mice

**Figure 9:**
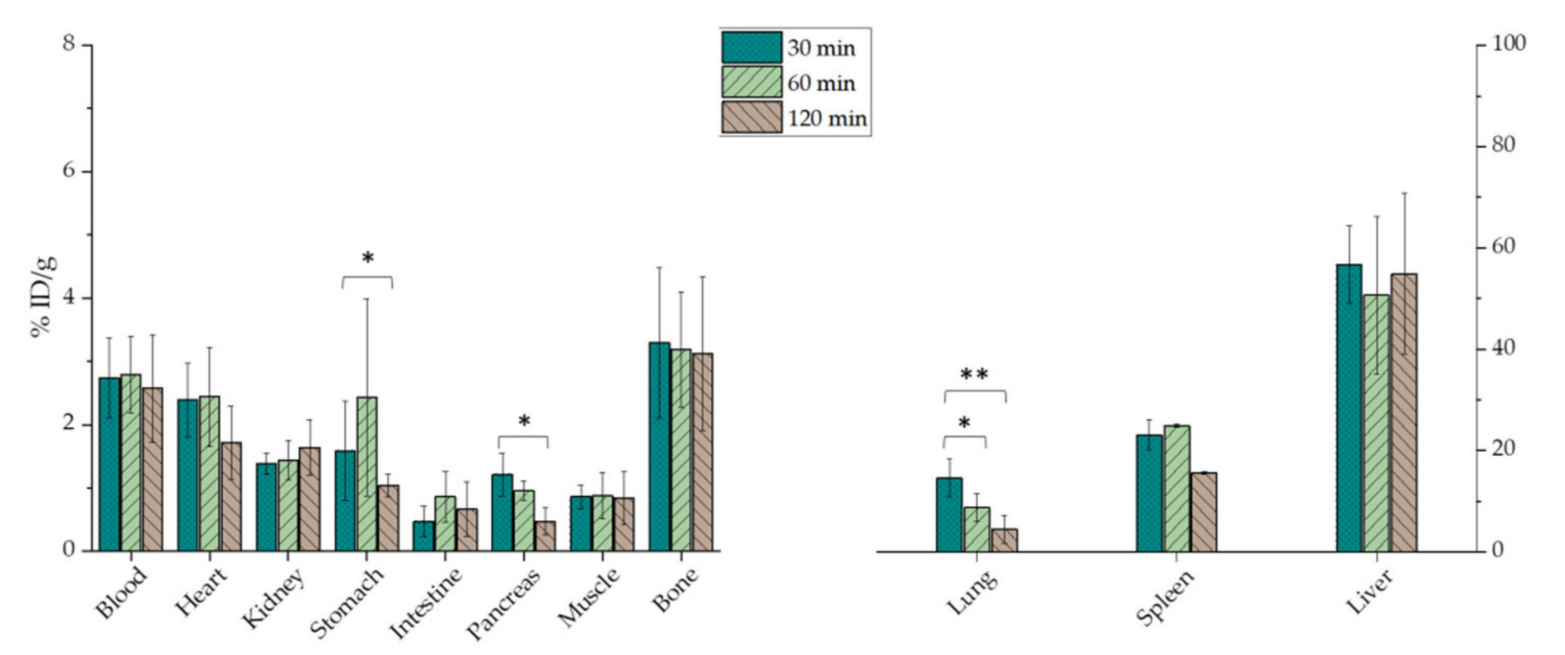
Biodistribution of [^68^Ga]Ga-MagP-NODAGA MIONs in normal mice (*n* = 3–5 mice per time point). Asterisks indicate the statistical significance of the difference between the results (* *p* < 0.05, ** *p* < 0.01, *** *p* < 0.001). Absence of asterisks denotes a nonsignificant statistical difference.

The biodistribution results, presented in Figure 9, show that the highest accumulation of [^68^Ga]Ga-MagP-NODAGA MIONs in normal mice is in the liver and spleen at all time points (indicatively, 54.86 ± 15.89% and 15.61 ± 0.27% at 120 min p.i., respectively). The uptake in lungs follows a decreasing pattern, falling from 14.58 ± 3.71% at 30 min p.i. to 4.51 ± 2.73% at 120 min p.i. Low bone accumulation is also observed, which remains stable throughout the time of study (3.12 ± 1.21%, 2 h p.i.). The retention of [^68^Ga]Ga-MagP-NODAGA MIONs in the bloodstream is practically constant, being 2.74 ± 0.63% at 30 min p.i. and 2.57 ± 0.85% at 120 min p.i. Finally, no major uptake of [^68^Ga]Ga-MagP-NODAGA MIONs in other organs is noted.

**Figure 10:**
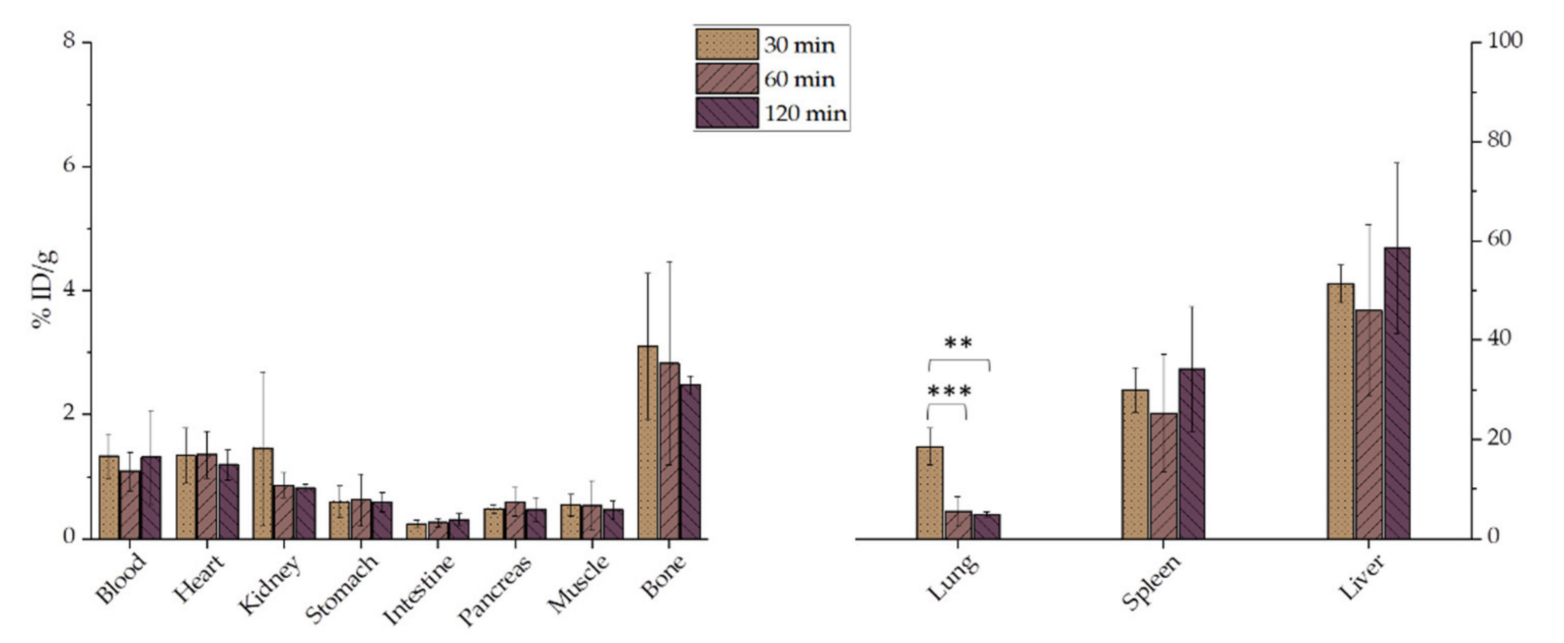
Biodistribution of [^68^Ga]Ga-MagP MIONs in normal mice (*n* = 3–5 mice per time point). Asterisks indicate the statistical significance of the difference between the results (* *p* < 0.05, ** *p* < 0.01, *** *p* < 0.001). Absence of asterisks denotes a nonsignificant statistical difference.

Regarding [^68^Ga]Ga-MagP MIONs, accumulation in the liver and spleen (58.55 ± 17.19% and 34.20 ± 12.52%, 120 min p.i., respectively) does not vary significantly between time points, whereas in the lungs there is a declining trend, similar to [^68^Ga]Ga-MagP-NODAGA MIONs (Figure 10). The presence of [^68^Ga]Ga-MagP MIONs in blood is stable (indicatively, 1.32 ± 0.75% at 120 min p.i.). The biodistribution behavior of [^68^Ga]Ga-MagP in the remaining organs was similar to that of [^68^Ga]Ga-MagP-NODAGA MIONs.

It has been noted that the biodistribution of nanoparticles is highly correlated with their physicochemical properties, such as size and surface chemistry [48,49]. After their intravenous administration, nanoparticles > 200 nm in size are rapidly covered by plasma proteins (opsonins), which are subsequently recognized by cells of the mononuclear phagocyte system (MPS), mainly Kupffer cells in the liver and macrophages in the spleen and bone marrow. As a result, these nanoparticles accumulate in the aforesaid organs, where they are phagocytosed [27,50]. On the other hand, ultrasmall nanoparticles are not recognized by MPS but are rapidly cleared by renal secretion [50,51]. Both opsonization and renal clearance can lead to decreased blood circulation, which compromises tumor uptake, hence the effectiveness of nanoparticles as cancer imaging agents. Another factor that affects the fate of nanoparticles is surface coating. In general, hydrophilic and neutral nanoparticle coatings contribute to the evasion of the MPS and thus to longer blood retention and a higher possibility for passive tumor targeting [36,37]. In our case, [^68^Ga]Ga-MIONs are mainly detected in the MPS organs of normal mice, primarily the liver and spleen. This fact can be attributed to their size and surface charge. [^68^Ga]Ga-MagP-NODAGA and [^68^Ga]Ga-MagP MIONs, with hydrodynamic diameters of 108 and 87 nm, respectively, follow the behavior of nanoparticles of similar size [5,52]. It is also reported that high negative nanoparticle surface charge may bring about more interactions with macrophages; thus, the surface charge of MIONs (−36 mV, at pH 7) may account for their high accumulation in MPS organs [30,53]. While the presence of the hydrophilic p(MAA-g-EGMA) coating resulted in their high colloidal stability in vitro, it did not inhibit uptake of these nanostructures in MPS organs in vivo, indicating that a higher molecular weight PEG is probably needed for effective steric stabilization of these MIONs in vivo. With regard to the excretion route, a minor accumulation in kidneys and a high liver and spleen uptake show that [^68^Ga]Ga-MIONs are mainly cleared by the hepatobiliary system and not by renal excretion. Overall, the in vivo behavior of [^68^Ga]Ga-MagP-NODAGA and [^68^Ga]Ga-MagP MIONs in normal mice is similar to the biodistribution of nanoparticles with comparable physicochemical properties [5,52,54]. Despite the high uptake of [^68^Ga]Ga-MIONs by the MPS organs, blood retention is satisfactory, which is encouraging for the continuation of studies in tumor-bearing mice.

#### 3.5.2. Ex Vivo Biodistribution Studies of [^68^Ga]Ga-MIONs in 4T1 Tumor-Bearing Mice

Studies in pathological mice were performed after the development of 4T1 tumors in SCID mice, namely on the 6th day after the subcutaneous injection of 1 × 10^6^ 4T1 cells in the front left limb of the animals.

**Figure 11:**
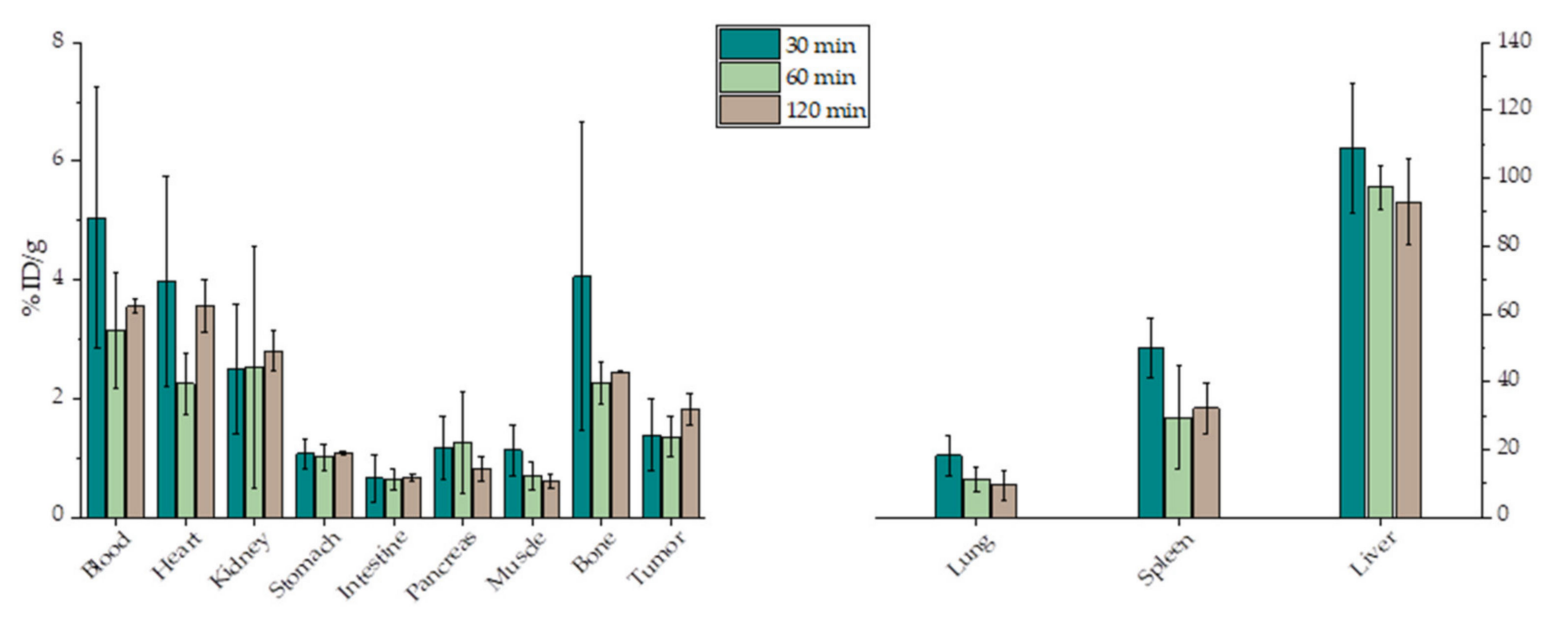
Biodistribution of [^68^Ga]Ga-MagP-NODAGA MIONs in SCID mice bearing 4T1 tumors (*n* = 3–5 mice per time point). Absence of asterisks denotes a nonsignificant statistical difference.

**Figure 12:**
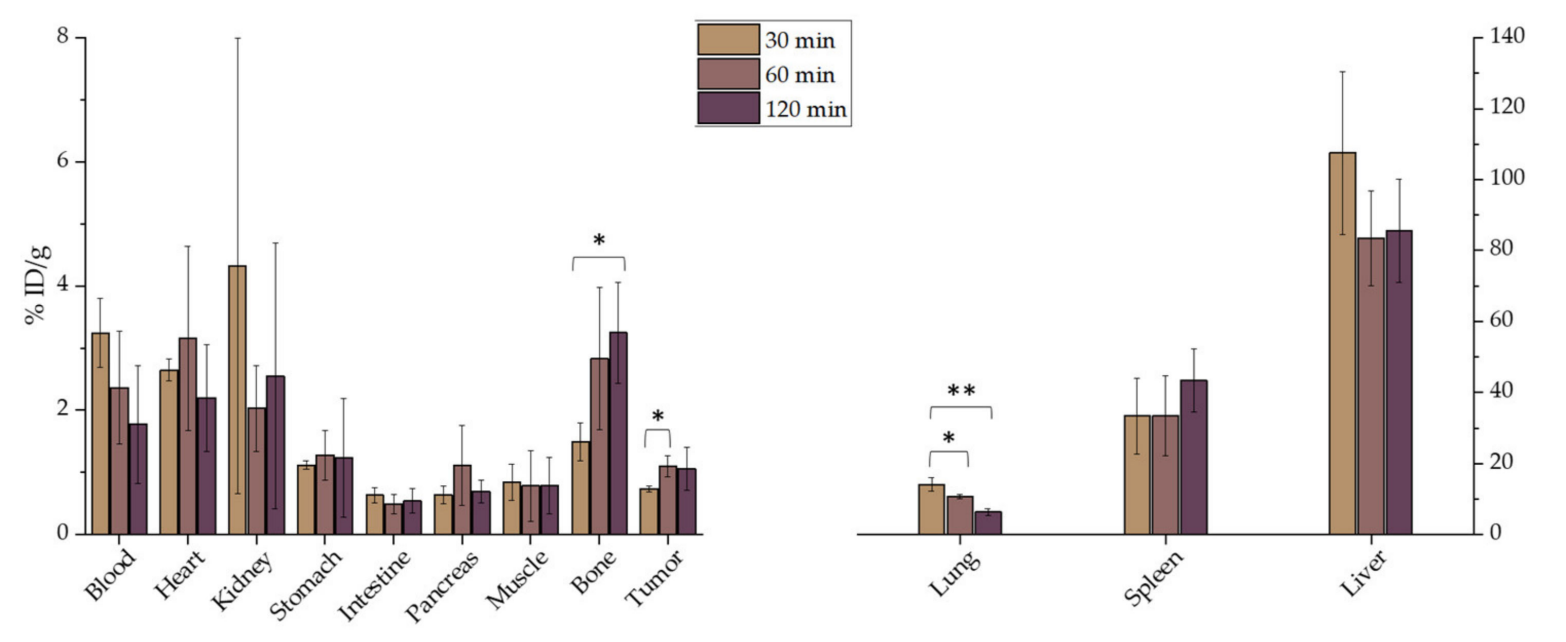
Biodistribution of [^68^Ga]Ga-MagP MIONs in SCID mice bearing 4T1 tumors (*n* = 3–5 mice per timepoint). Asterisks indicate the statistical significance of the difference between the results (* *p* < 0.05, ** *p* < 0.01, *** *p* < 0.001). Absence of asterisks denotes a nonsignificant statistical difference.

As expected, the biodistribution of both groups of nanoparticles in pathological mice follows a similar pattern to that in normal mice (Figure 11 and Figure 12). The organs with the highest accumulation throughout the time of study were the liver and spleen, followed by the lungs, where the uptake seems to decrease with time (the reduction was statistically significant in the case of [^68^Ga]Ga-MagP MIONs). In bones, the % ID/g of [^68^Ga]Ga-MagP-NODAGA MIONs is 4.05 ± 2.59% at 30 min and falls to 2.45 ± 0.01% at 120 min, while the uptake of [^68^Ga]Ga-MagP MIONs increases with time but practically reaches the same level with [^68^Ga]Ga-MagP-NODAGA MIONs at 120 min p.i. (3.24 ± 0.82%). Radiotracer uptake in the kidneys, as well as in the other organs, is minor in both groups.

The accumulation of both [^68^Ga]Ga-MIONs in the tumor follows an increasing trend, i.e., the % ID/g of [^68^Ga]Ga-MagP-NODAGA MIONs is 1.39 ± 0.60% at 30 min p.i. and 1.83 ± 0.27% at 120 min p.i., while for [^68^Ga]Ga-MagP, the observed tumor uptake was 0.74 ± 0.05% and 1.06 ± 0.35% at 30 and 120 min p.i., respectively (a statistically significant increase was found in the case of [^68^Ga]Ga-MagP MIONs, as shown in Figure 12).

A factor to be considered during the evaluation of a cancer imaging agent candidate is the uptake ratio of tumor to organs or tissues (signal-to-noise ratio) because it contributes to image quality. Specifically, tumor/blood and tumor/muscle ratios are important because blood and muscle are ubiquitous in the body, and low tumor/tissue ratios can compromise image quality. In the case of [^68^Ga]Ga-MagP-NODAGA MIONs, the tumor/blood ratio at 30 min. p.i. was 0.275 ± 0.002, while the respective ratio for [^68^Ga]Ga-MagP MIONs was 0.23 ± 0.03. The accumulation of both complexes in the tumor is higher compared to that in the muscles at all time points (except for the [^68^Ga]Ga-MagP MIONs at 30 min p.i., where the two uptakes are almost equal). Accordingly, the tumor/muscle ratios are equal to or above unity, which is a positive feature for bioimaging applications. 

Overall, the biodistribution and tumor uptake of [^68^Ga]Ga-MIONs in tumor-bearing mice is in good agreement with results by other groups. As reported, biopolymer-based nanoparticles of ~110 nm in size and with negative surface charge have a differential absorption ratio less than 1 in nonspecific tumor targets [55]. In another study, magnetic nanoparticles of similar size, grafted with p(MAA-g-EGMA) showed accumulation of 0.8–0.9% ID/g in a tumor xenograft after their intravenous administration [44].

A factor that may affect the tumor uptake of [^68^Ga]Ga-MIONs is the cancer model studied [17]. It is thought that passive targeting depends on the intrinsic tumor biology, namely different tumor types may differentiate in their extent of hypervasculature or vessel architecture [56]. Future studies including tumors other than 4T1 are within our goals.

Several studies propose that the decorating of nanostructures with targeting molecules presents improved tumor retention [30,57]. Thus, the functionalization of MIONs with ligands that recognize specific tumor targets could grant higher tumor accumulation to provide a better imaging performance.

#### 3.5.3. In Vivo Imaging Studies (Pilot Studies)

**Scheme 1:**
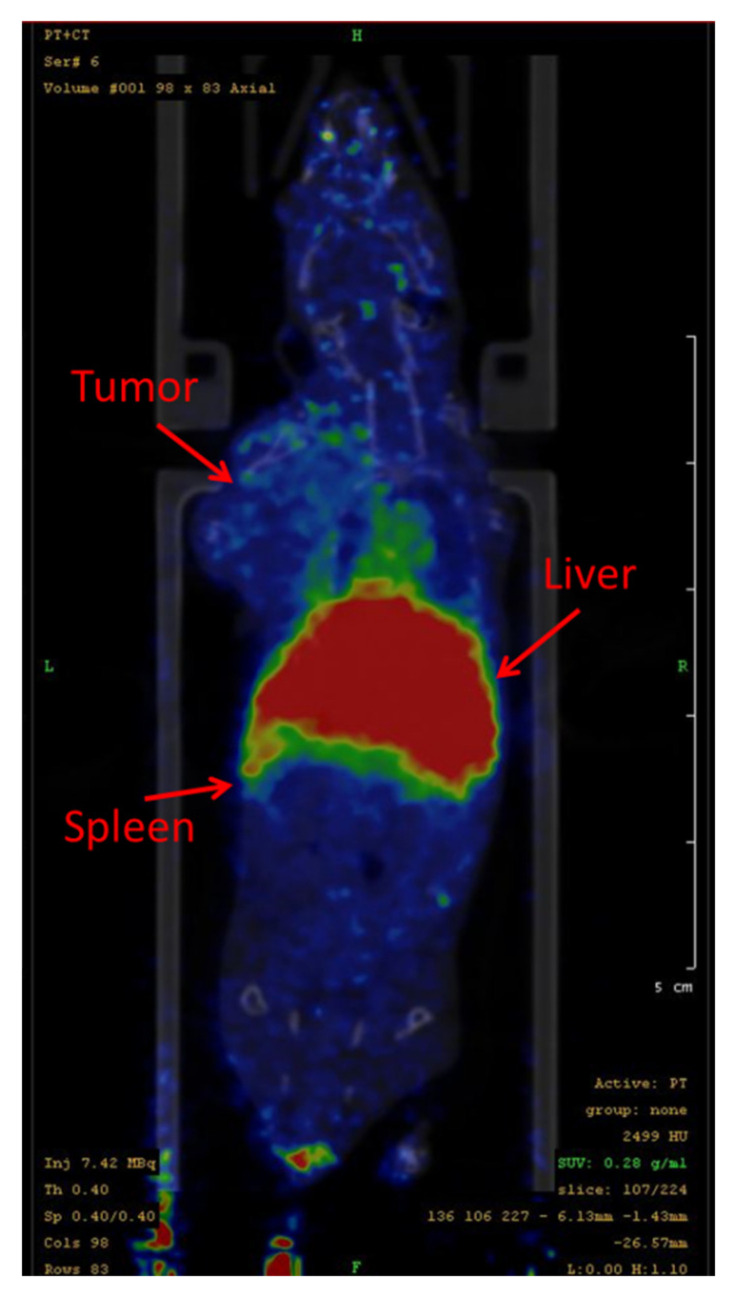
PET image of a 4T1 tumor-bearing SCID mouse injected with [^68^Ga]Ga-MagP MIONs at 120 min p.i.

After evidence of a degree of tumor accumulation was provided by the ex vivo biodistribution study, a pilot PET imaging study was performed on a 4T1 tumor-bearing SCID mouse to further evaluate the tumor accumulation of the developed radiolabeled nanoconstructs. The PET image of a mouse injected with [^68^Ga]Ga-MagP MIONs showed radiotracer uptake in the tumor, while the main organs of accumulation were the liver and spleen (Scheme 1). These results are in direct agreement with the ex vivo biodistribution results initially performed in tumor-bearing mice (Figure 11 and Figure 12). In vivo assessment on the PET camera after 120 min p.i. of the radiotracer was not possible, due to the short half-life of ^68^Ga.

## 4. Conclusions

Magnetic iron oxide nanoparticles were successfully synthesized and surface functionalized with the poly(methacrylic acid)-graft-poly(ethyleneglycol methacrylate) copolymer (p(MAA-g-EGMA)), providing colloidal stability and biocompatibility of MIONs, while at the same time exhibiting the advantage of the diversity of choice for radiolabeling with ^68^Ga. In this study, two different approaches were explored to develop ^68^Ga-labeled MIONs. For the chelator-mediated approach, the chelating agent NODAGA-NHS was conjugated onto the surface of the MIONs (MagP-NODAGA) to form a chelate complex with ^68^Ga. In the chelator-free approach, ^68^Ga was directly incorporated onto the surface of the MIONs (MagP). Both approaches succeeded in the rapid and highly efficient labeling of MIONs with ^68^Ga, and stable conjugates were formed. Although ex vivo biodistribution studies of [^68^Ga]Ga-MIONs showed high accumulation in the MPS organs, they indicated satisfactory blood retention at 30, 60, and 120 min p.i. Furthermore, the tumor/muscle ratios were all above 1. In vivo PET imaging of a mouse injected with [^68^Ga]Ga-MagP MIONs showed that the results are in direct agreement with the ex vivo biodistribution results initially performed in tumor-bearing mice. Finally, the low toxicity of MagP and MagP-NODAGA MIONs against 4T1 breast cancer cells gives an indication of their in vivo biocompatibility. These preliminary studies established that [^68^Ga]Ga-MIONs exhibit potential for bioimaging applications. 

## Data Availability

The data presented in this study are available upon request from the corresponding author P.B.
